# Examining the Current and Future Scientific Field of Antidoping: “Cheaters Should Never Win”

**DOI:** 10.3389/fspor.2020.596815

**Published:** 2020-10-08

**Authors:** Raphael Faiss, David Pavot

**Affiliations:** ^1^REDs, Research & Expertise in Anti-doping sciences, University of Lausanne, Lausanne, Switzerland; ^2^Research Chair on Anti-doping in Sports, Department of Marketing, Business School, University of Sherbrooke, Sherbrooke, QC, Canada

**Keywords:** antidoping, deterrence, detection, enforcement, rule of law, performance, ergogenic

To frame the current advances in anti-doping sciences, an initial definition of doping is necessary while it may in all cases foster a lively debate. The 2021 World Anti-Doping Code defines doping in Article 1 as “one or more of the anti-doping rule violations set forth in Articles 2.1 to 2.11 of the Code” (WADA, 2019) with an extremely detailed “Prohibited List” covering the Use or Attempted Use of doping substances and methods and certain malicious practices (Pavot, 2020). More simplistically, antidoping provisions may be considered violated when an athlete uses or attempts to use a prohibited substance or method or when a prohibited substance is detected in an urine or blood sample. Much then relies on the technical ability of an antidoping laboratory to detect such method or substance within a strict scope of international standards and operating guidelines. In an ideal scenario, laboratories would define and disseminate standard testing procedures for all kind of existing and upcoming substances, with unequivocal criteria for the definition of positivity, and the procedures would have been previously validated in blinded randomized and controlled studies with athletic subjects from both sex. Moreover, the epitome of experiments would make the sanctioning process swift with undeniable definitions of substances, dose and timing of use, administration, and individual metabolic variations (Faiss et al., 2019).

In the current world of global sports, the context is much more complex with each sporting performance scrutinized, and criticized often with a distorted judgement. The fight against doping is today at a crossroads. Multidisciplinary issues at stake in the social, biological, and global sciences should enable the system to move forward so that cheaters never win—or at the very least—their victory is increasingly difficult and risky through the threat of control, denunciation, or otherwise.

In this context, the contribution of non-biological disciplines also appears fundamental throughout the process. Education is a critical issue since the culture of the fight against doping must be instilled in athletes and their entourage from an early age. Moreover, the sanction-based argument is now outdated and a new argument, based on the values of clean sport, must be supported. However, these programs are often implemented by National Antidoping Organizations (NADOs) who face multiple challenges in making them efficient (Gatterer et al., 2020).

Other topics demonstrate the usefulness of the contribution of disciplines such as law, political science, communication, and even marketing. Obviously, with the importance of the World Anti-Doping Code and disputes before the Court of Arbitration for Sport, the importance of the legal field seems natural. Political and governance issues have received renewed interest in recent years: one can think, for example, of the concerns raised by the Office of National Drug Control Policy (ONDCP) Report of 17 June 2020 to the U.S. Congress regarding WADA Reform Efforts, which suggested, among other things, that the U.S. financial contribution to WADA be suspended. This report focuses—albeit in a biased way—on the issues at stake in the governance reform of WADA that is currently underway as well as on the major political issues that are currently at stake in the fight against doping. We could also talk about the relationship between WADA and the IOC, NADOs, and international federations, the question of gender representativeness in antidoping bodies, etc. Communication and marketing issues are also at the heart of the questioning. A certain lack of knowledge about WADA's functions sometimes raises many expectations that it cannot meet. WADA is more of a regulator than a player, and the media are sometimes unaware of its role. Moreover, the 2020–2024 strategic plan is not mistaken in this regard, since a project on the agency's brand image is clearly mentioned.

The question of whistleblowers is a particularly topical issue. The World Anti-Doping Agency (WADA) has launched the *Speak Up!* platform to encourage whistleblowers to come forward, but its implementation must be accompanied by protection for whistleblowers, who sometimes risk their lives to report such actions. However, not all States necessarily play the game, as is shown by the deadly Rodchenkov Act (Haas, 2020) in the United States, which provides—not for the protection of potential whistleblowers—but for universal criminal jurisdiction to be granted to the United States if it has been involved in doping activities in the past. While penalizing doping may be an avenue, albeit somewhat outdated, the protection of whistle-blowers to dismantle important—sometimes transnational or even State owned—networks seems to be a higher interest. However, the Rodchenkov act seems to be the opposite of what should be done.

One can also think of the protection of anti-doping organizations as an example of multidisciplinarity in the social sciences. On June 15, 2018, the Parliament of the Province of Quebec adopted Bill 238 concerning immunities granted to the World Anti-Doping Agency. Immunities are used to protect an organization from prosecution or seizure in the country that grants them. During the examination of the bill in the Parliamentary Committee, Olivier Niggli, the Director General of WADA, even qualified the Agency as a “special animal” with regard to its status. Indeed, the WADA is a foundation under Swiss law which is not an international organization but an international non-governmental organization. After the exclusion of Russia from the Rio Olympic Games after a thorough investigation, WADA has been subjected to several reactions. In September 2016, the Russian “Fancy Bears” hacker group entered the WADA's servers, revealing a series of confidential information relating in particular to the therapeutic use exemptions used by some of the best-known athletes. The Canadian authorities, WADA's host country, decided to investigate that specific situation and concluded that cybersecurity measures should be proportionate both to the sensitivity of the personal information being protected and to the attractiveness of the information to malign actors. Parallel to this investigation, the WADA and Richard McLaren, author of the eponymous report on Russia, were sued in the Ontario Superior Court. The evolution of WADA's functions and these two events led WADA to negotiate the granting of immunity, which was favorably received by Quebec, where WADA's head office is located. This is an exemplary collaboration between the legal and political communities. More broadly, this example highlights the need for specific treatment of national and international agencies involved in the fight against doping and their staff. When the Russian authorities threaten to imprison any independent investigator who sets foot on its territory, it is the system that must change.

The current developments and biggest challenges in the fight against doping may indeed also be illustrated by the example of blood doping and its detection. Performance in sports relies heavily on the body's ability to produce as much power with a very high convective transport of oxygen to the working muscles. Adequate training (sometimes at altitude) or blood transfusions are hence known to improve performance. The use of blood transfusions was widespread in athletics until the Olympic Games in Los Angeles in 1984, since the method was not prohibited although it proved to be very effective in increasing performance. The ban issued by the International Olympic Committee (IOC) in 1986 only partially resolved the situation as this method remains very difficult to detect. A few years later (1989), erythropoietin (EPO) was firstly commercialized and subsequently banned in sports but adopted by cheaters as a precursor to the production of blood cells (erythrocytes) in the bone marrow. Since then, many elite athletes have abused recombinant human erythropoietin (rhEPO) for its remarkable effect on physical performance and its widespread availability on the market for sports drugs. In the absence of a detection method in the 1990s, its use was even endemic in endurance sports such as cycling, Nordic skiing, or athletics. For example, it is interesting to note a more marked improvement in the best annual world performances over 10,000 m from 1989 (Iljukov and Schumacher, 2017). There is thus certainly a link between the accessibility of ergogenic products, the development of methods for detecting these products and ultimately their antagonistic effects on sports performance. Following the implementation of a direct EPO detection method developed timely for the 2000 Sydney Olympics (Saugy and Leuenberger, 2020), athletes turned to new generations of rhEPO that were quickly included in the EPO detection spectrum. With detection methods becoming more sensitive, athletes began to adjust their doping regimen. By adjusting the route and time of administration to favor intravenous injections of first generation rhEPO (with a shorter half-life) just before bedtime, athletes reduce the risk of their doping being detected, such as sampling cannot take place in the middle of the night. Notably, the Court of Arbitration for Sport has systematically validated the sanctions linked to the use of the EPO detection method to identify the presence of EPO of exogenous origin in the urine of cheaters.

In parallel with the direct detection of EPO, the idea of a longitudinal follow-up of biological variables of the athlete largely developed in the 2000s. Robust blood parameters (the level of red blood cells or hematocrit in this case) were already measured for anti-doping purposes, and some federations (the International Ski Federation and the International Cycling Union) implemented a “no-start” rule for athletes with a hematocrit >50%. This measure saw spectacular effects, notably with the exclusion of the leader from the Giro on the eve of the arrival in 1999. The use of blood markers for anti-doping purposes then evolved with the development of the “OFF-Score” as a stimulation index of the reticulocytes (or the “young” red blood cells) (Parisotto et al., 2000). However, the interpretation of secondary blood markers can become hazardous in the case of discontinuous rhEPO treatment (Robinson et al., 2002). The feasibility of longitudinal monitoring of hematological values for endurance sports athletes was then evaluated (Malcovati et al., 2003). In the process, the statistical definition of what an abnormal blood profile represents (Sottas et al., 2006) precedes the concrete development of an “Athlete Biological Passport” (ABP) at the Swiss laboratory for doping analysis in Lausanne as a method of indirect detection of blood doping (Sottas et al., 2010). The ABP was initially used in 2009 in cycling and officially adopted by the World Anti-Doping Agency (WADA) in December of the same year.

The example of blood doping illustrates a change in paradigm in favor of the ABP with a strong contributions of scientific research to the fight against doping. Developing innovative techniques is necessary to promote an enforcement of the rule with applicable sanctions. “Omics” approaches sound promising for example, with metabolomics for the detection of steroids (Ponzetto et al., 2016) but also for the detection of EPO (Appolonova et al., 2008). However, the main disadvantage of these studies with a lack of decisive discoveries (Faiss et al., 2019) lies in the limited number of data, because most of the time a targeted methodology was applied and only a few samples could be analyzed. Conversely, recent research may further tighten a net to catch dopers with the detection of blood doping in urine (Bejder et al., 2020) or blood (Malm et al., 2020) with an extended detection window.

In addition, recent advances in detection methods (i.e., mass spectrometry) now make it possible to detect thousands of compounds involved in various molecular pathways. The biomarkers derived from these data could constitute a powerful complement to those used in ABP. However, the obstacle of the cost of these methods and the understanding (or translation) for lawyers of the scientific elements that could lead to a decision on a potential sanction remains to be overcome.

Finally, the state of research demonstrates the need to decompartmentalize disciplines between the humanities and the biological sciences. The first and most blatant example is that of legal challenges to sanctions, in particular before the Court of Arbitration for Sport. For years now, sanctions have been contested by means of lawyers, but the parties to disputes also resort to scientific expertise to validate or discredit certain hypotheses demonstrating the weight of science in legal arguments. In recent times, some cases have also received a lot of media coverage, and some have even been fought on social networks, such as the Sun Yang case. A less known example is the sharing of information between the various national and international actors involved in the fight against doping. While the system appears to work for testing in terms of methods for searching for substances or distribution plans, the same cannot be said for education or surveys. In education, greater coordination of initiatives would certainly allow for economies of scale in order to avoid redundant practices. Finally, in the case of investigations, several factors explain a more relative cooperation: the culture of secrecy, the involvement of various national and international authorities, the weight of bureaucracy or even legislative or regulatory provisions that do not allow such sharing, however crucial it may be.

It should be remembered, however, that athletes who abuse doping substances do so to trigger physiological changes leading to physiological improvements. So, despite remarkable results in its implementation, athletes are getting used to fine-tuning their doping methods to bypass the current rules. Finally, if the World Anti-Doping Code is getting thicker to exclude more exceptions to the rules, appropriate research in Anti-Doping sciences will contribute to solve individual cases. The evolution of antidoping rules against blood doping illustrates for example almost 30 years of scientific developments toward strong methods with a high discriminative power and unequivocal results. One may see the loopholes in the latter development with counterfeits adapting rapidly or the opportunities left for decisive discoveries bridging the gap behind the cheaters. Definitely, education, deterrence, detection, enforcement, and rule of law represent five firm pillars to delineate a broad field of exploration of doping and anti-doping initiatives. We strongly believe that antidoping sciences may contribute to bring such decisive breakthroughs or at least nourish the lively debate on how to better outline doping and fight it.

## Author Contributions

RF and DP drafted the manuscript. All authors contributed equally to the final version and expressed their approval of the final submitted version.

## Conflict of Interest

The authors declare that the research was conducted in the absence of any commercial or financial relationships that could be construed as a potential conflict of interest.
